# Supplementary material for: MetaRibo-Seq measures translation in microbiomes
Source: Nat Commun. 2020 Jun 29;11:3268. doi: 10.1038/s41467-020-17081-z (PMC7324362; doi:10.1038/s41467-020-17081-z)
Supplement: Supplementary file 10 — Supplementary Data 7 [file 41467_2020_17081_MOESM10_ESM.zip › File2/Confidence_High_Taxonomy/319796_out.krona.html]

Javascript must be enabled to view this page.

members
magnitude
magnitudeUnassigned
count
unassigned
taxon
rank

319796\_out

55

2
55
superkingdom

976
55
phylum

200643
class
55

order
55
171549

51
family
171552

genus
49
838


SRS013521\_contig\_number\_581SRS144537\_contig\_number\_38715
1263102
2
species

59823

SRS045713\_contig\_number\_12226SRS047433\_contig\_number\_11145SRS049712\_contig\_number\_16051SRS077641\_contig\_number\_4577SRS143876\_contig\_number\_4392SRS144297\_contig\_number\_8607SRS144506\_contig\_number\_23010SRS144603\_contig\_number\_5149
8
species

165179

SRS011134\_contig\_number\_31594SRS013638\_contig\_number\_10196SRS013687\_contig\_number\_13824SRS014415\_contig\_number\_554SRS016541\_contig\_number\_9011SRS017375\_contig\_number\_6SRS018888\_contig\_number\_682SRS019285\_contig\_number\_86SRS022609\_contig\_number\_19641SRS043667\_contig\_number\_5621SRS043841\_contig\_number\_1255SRS044535\_contig\_number\_11129SRS046502\_contig\_number\_13633SRS047044\_contig\_number\_7316SRS049896\_contig\_number\_9433SRS049995\_contig\_number\_6991SRS053356\_contig\_number\_38897SRS064757\_contig\_number\_13960SRS065397\_contig\_number\_1985SRS074586\_contig\_number\_1101SRS077194\_contig\_number\_14021SRS077849\_contig\_number\_20398SRS077991\_contig\_number\_67SRS078176\_contig\_number\_11262SRS078419\_contig\_number\_3230SRS103987\_contig\_number\_4035SRS104036\_contig\_number\_6519SRS1041031\_contig\_number\_17538SRS1041116\_contig\_number\_821SRS1041134\_contig\_number\_2804SRS1041137\_contig\_number\_13715SRS1041140\_contig\_number\_9154SRS104327\_contig\_number\_35SRS105082\_contig\_number\_12679SRS146764\_contig\_number\_23677SRS147614\_contig\_number\_532SRS148253\_contig\_number\_3453SRS150029\_contig\_number\_11114
species
38

1
species

SRS012969\_contig\_number\_12941
2292365

genus
2
577309

454154

SRS015782\_contig\_number\_3453SRS017307\_contig\_number\_8082
species
2

815
4
family

816
genus

SRS144714\_contig\_number\_24964
1
4

1
species

SRS023715\_contig\_number\_10688
357276

1
species

SRS053398\_contig\_number\_7913
28116


SRS097958\_contig\_number\_10622
246787
1
species
